# Phylogenetic relationships and antimicrobial susceptibility of human- and parrot-derived *Chlamydia psittaci* isolates in Shenzhen, China

**DOI:** 10.3389/fcimb.2026.1836823

**Published:** 2026-07-08

**Authors:** Wenkai Cui, Shuang Wu, Qiongcheng Chen, Xinyue Lei, Huiqun Lu, Shiting Chen, Dongfeng Kong, Shousheng Lu, Liyang Wu, Ming Jiang, Li Yuan, Jianguang Cai, Xiaolu Shi

**Affiliations:** 1 School of Public Health, University of South China, Hengyang, China; 2 Shenzhen Center for Disease Control and Prevention, Shenzhen, China; 3 Liuzhou Center for Disease Control and Prevention, Guangxi, China; 4 School of Public Health, Shanxi Medical University, Taiyuan, China; 5 Guangdong Center for Animal Disease Control, Guangzhou, China; 6 School of Public Health, Southern Medical University, Guangzhou, China

**Keywords:** antimicrobial susceptibility, *Chlamydia psittaci*, psittacosis, whole-genome sequencing, zoonotic transmission

## Abstract

**Introduction:**

Chlamydia psittaci is an important zoonotic pathogen mainly transmitted to humans through exposure to infected birds or contaminated bird excreta, and infection may range from mild respiratory illness to severe systemic disease.

**Method:**

Between 2023 and 2024, 114 samples were collected in Shenzhen, China, including bronchoalveolar lavage fluid (BALF) samples from four laboratory-confirmed psittacosis patients, five tissue samples from one suspected infected parrot, and 105 oropharyngeal and cloacal swabs from parrots in a local bird market. C. psittaci was detected by conventional PCR targeting ompA. Positive samples were subjected to targeted capture sequencing, isolation in McCoy cells, ompA-based genotyping, whole-genome SNP phylogenetic analysis, and intracellular antibiotic inhibition assays.

**Results:**

C. psittaci DNA was detected in 42 samples, of which 19 yielded high-quality genomic sequence data. Three isolates were successfully cultured: SZ1 from parrot trachea, SZ2 from parrot small-intestinal mucosa, and SZ3 from BALF from Case 1. Phylogenetic analyses based on ompA and genome-wide SNPs showed that all three isolates belonged to genotype A and clarified their relationships with previously reported strains. No known acquired antimicrobial resistance genes were identified, but nucleotide substitutions were detected at selected loci previously reported to be potentially associated with antimicrobial susceptibility. Comparative intracellular inhibition assays showed different antibiotic response patterns between the parrot-derived isolate SZ1 and the human-derived isolate SZ3; SZ1 showed a less pronounced inhibitory response to doxycycline under the present in vitro conditions, suggesting reduced doxycycline susceptibility rather than confirmed resistance.

**Discussion:**

These findings highlight the value of genomic surveillance and antimicrobial susceptibility monitoring of C. psittaci isolates from both avian reservoirs and human infections, which may support risk assessment and clinical management of psittacosis.

## Introduction

*Chlamydia psittaci* (*C. psittaci*) is a widely distributed obligate intracellular zoonotic pathogen that infects both birds and mammals, causing avian chlamydiosis in birds and psittacosis in humans (Huang et al., 2023; Wang et al., 2024). In birds, infection may be asymptomatic or may manifest as respiratory, digestive, or systemic disease, and infected birds can intermittently shed the pathogen through respiratory secretions and feces. Psittacine birds, pigeons, ducks, turkeys, and other domestic or wild birds have been recognized as important reservoirs or sources of human infection. Humans are infected mainly through inhalation of aerosolized particles derived from dried feces, respiratory secretions, contaminated feathers, or environmental dust from infected birds. Less commonly, infection may occur through direct contact with infected birds, including bites or beak-to-mouth contact (Ayllón et al., 2025).

The prevalence of avian chlamydial infection varies substantially across host species, geographic regions, sampling strategies, and detection methods. A global meta-analysis reported that the prevalence of chlamydial infections in birds has remained around 20% in recent years, with differences between live specimens and carcass specimens as well as between PCR-based and non-PCR-based detection methods (Sukon et al., 2021). Studies from China have also reported variable positivity rates among different bird populations and settings, including poultry, pigeons, pet birds, and live-bird markets, indicating that *C. psittaci* may persist in bird-associated environments and pose a potential zoonotic risk (Zhang et al., 2024).

In humans, psittacosis may present as a self-limiting febrile respiratory illness but can also progress to severe systemic disease, underscoring its public health relevance (Zeng et al., 2025). The disease is frequently associated with occupational or environmental exposure to infected birds, including pet-bird owners, bird breeders, veterinarians, poultry workers, and workers in live-bird markets or slaughterhouses. Because infected birds may be asymptomatic while still shedding the pathogen, surveillance at the human–bird interface is important for early detection and prevention of zoonotic transmission.

Previous genomic studies have demonstrated substantial genetic diversity within *C. psittaci*, with distinct *ompA*-based genotypes associated with different host species and transmission patterns (Anstey et al., 2021). Whole-genome sequencing has therefore become an important tool for investigating the molecular epidemiology, evolutionary relationships, and potential host adaptation of *C. psittaci* strains (Kasimov et al., 2023). However, evidence regarding antimicrobial resistance or reduced antimicrobial susceptibility in *C. psittaci* remains limited, particularly for strains circulating at the human–bird interface in urban environments. This is clinically relevant because doxycycline remains the first-line treatment for psittacosis, while antimicrobial use in bird-associated settings may influence susceptibility patterns over time.

Therefore, the present study aimed to investigate the occurrence, genotypes, genomic characteristics, and antimicrobial susceptibility profiles of *C. psittaci* strains detected in human clinical and parrot-associated samples in Shenzhen, China. By combining molecular detection, isolation, *ompA*-based genotyping, whole-genome single-nucleotide polymorphism analysis, and intracellular antibiotic inhibition assays, this integrated approach was designed to provide evidence for genomic surveillance and antimicrobial susceptibility monitoring of *C. psittaci* at the human–bird interface.

## Materials and methods

### Sample collection

Samples were collected in Shenzhen, China, between March 2023 and May 2024. A total of 114 samples were included in this study, comprising four bronchoalveolar lavage fluid (BALF) samples from laboratory-confirmed psittacosis patients, 105 swab samples collected from parrots in a wholesale bird market in Futian District, Shenzhen, and five tissue samples collected from one suspected infected parrot. The 105 parrot swab samples consisted of 65 cloacal swabs and 40 oropharyngeal swabs. The five tissue samples consisted of trachea, small-intestinal mucosa, lung, liver, and kidney and were processed individually rather than pooled.

Oropharyngeal and cloacal swabs were collected directly from parrots in the bird market. Each sample was labeled according to cage number and on-site epidemiological investigation records, allowing PCR-positive samples to be linked to the corresponding cages and birds. One suspected infected parrot was selected for veterinary diagnostic necropsy based on epidemiological investigation, cage information, and PCR screening results. Five tissue samples, including trachea, small-intestinal mucosa, lung, liver, and kidney, were collected from this parrot and processed individually. No parrot was euthanized solely for the purpose of this study. Tissue sampling was performed during veterinary diagnostic necropsy under approved animal ethics procedures.

The use of human clinical samples was approved by the Ethics Committee of the Shenzhen Center for Disease Control and Prevention (approval no. SZCDC-IRB2026042), and written informed consent was obtained from all patients. Animal sampling and necropsy procedures were performed in accordance with institutional animal ethics guidelines and were approved by the Animal Ethics Committee of the Shenzhen Center for Disease Control and Prevention (approval no. SZCDC-IRB2026042).

### Polymerase chain reaction assays and sequencing

BALF samples were directly subjected to DNA extraction. Oropharyngeal and cloacal swab samples were vortexed thoroughly in sterile phosphate-buffered saline, and the resulting suspension was used for DNA extraction. Tissue samples from the suspected infected parrot were homogenized separately in sterile phosphate-buffered saline and centrifuged at low speed to remove tissue debris; the supernatant was then used for DNA extraction and cell culture inoculation. DNA was extracted using the QIAamp DNA Mini Kit (QIAGEN, Hilden, Germany) according to the manufacturer’s instructions. Different PCR-based assays were used for different purposes in this study. Conventional PCR targeting the *ompA* gene was used for initial screening of *C. psittaci*-positive samples and for subsequent genotyping. Quantitative real-time PCR (qPCR) targeting *ompA* was used to assess bacterial proliferation during isolation. Reverse transcription quantitative PCR (RT-qPCR) targeting *ompA* was used in the intracellular antibiotic inhibition assays to evaluate changes in bacterial replication after antibiotic exposure.

Screening for *C. psittaci* was performed using a previously described conventional PCR assay targeting the *ompA* gene, which yields an expected amplicon of approximately 1050 bp (Stenzel et al., 2014). Briefly, each 25-μL reaction contained 12.5 μL of 2× PCR Master Mix, 0.5 μM of each primer, and 2 μL of template DNA. Thermal cycling consisted of an initial denaturation at 95 °C for 5 min, followed by 35 cycles of 95 °C for 30 s, 55 °C for 30 s, and 72 °C for 1 min, with a final extension at 72 °C for 7 min. Samples showing the expected amplification band were considered PCR-positive and were subsequently selected for targeted capture sequencing.

A total of 42 samples were positive for *C. psittaci* DNA by conventional PCR and were selected for targeted capture sequencing. Targeted capture sequencing was used as an enrichment-based approach to improve the recovery of pathogen-derived DNA from samples with low bacterial loads. Nineteen sequencing datasets, including 16 direct sample-derived datasets and three cultured-isolate datasets, yielded genomic data of sufficient quality for downstream genotyping and comparative genomic analyses. The 16 direct sample-derived datasets were generated by iGeneTech Biotechnology (Beijing, China), and the three cultured-isolate datasets were generated by Nuohe Biotechnology (Beijing, China).

Target enrichment was performed using a custom hybridization capture panel developed by iGeneTech Biotechnology (Beijing, China). The panel consisted of 100-bp biotinylated DNA probes designed from conserved genomic regions identified among 25 publicly available *C. psittaci* reference genomes downloaded from the NCBI database. Probe deduplication was performed to reduce redundancy in highly conserved regions and to improve the uniformity of capture depth across the genome. The probes were used to enrich pathogen-derived DNA fragments prior to sequencing.

Sequencing libraries were prepared using the IGT^®^ Enzyme Plus Library Prep Kit V3 (iGeneTech, Beijing, China), and hybridization capture was performed using the TargetSeq One^®^ Hyb & Wash Kit v2.0 (iGeneTech, Beijing, China) according to the manufacturer’s instructions. Enriched libraries were sequenced on an Illumina NovaSeq 6000 platform to generate 2 × 150 bp paired-end reads. Downstream read processing, host-read removal, genome assembly, consensus genome generation, and variant calling are described in the Genomic analysis section.

### Isolation and identification of *C. psittaci*

Isolation was attempted from PCR-positive BALF and tissue samples, including tracheal and small-intestinal mucosal tissues from the suspected infected parrot, using McCoy cell cultures according to previously published methods (Pearson et al., 1989). Successful isolates were obtained from one tracheal tissue sample, one small-intestinal mucosal tissue sample, and one BALF sample, corresponding to SZ1, SZ2, and SZ3, respectively.

McCoy cells were maintained in Dulbecco’s modified Eagle medium (DMEM) supplemented with 10% fetal bovine serum and 1% actinomycin at 37 °C in a humidified atmosphere containing 5% CO_2_. Vancomycin (25 μg/mL) and gentamicin (10 μg/mL) were added to suppress bacterial contamination during isolation. Inoculated cells were monitored and blindly passaged three times.

qPCR targeting the *ompA* gene of *C. psittaci* was performed using previously described primers (Mitchell et al., 2009) to compare bacterial loads between the original samples and cultured materials, thereby assessing pathogen proliferation during cell culture. Successful isolation was confirmed by qPCR, Wright–Giemsa staining, immunofluorescence staining, and transmission electron microscopy (TEM).

Infected McCoy cells were examined by Wright–Giemsa staining at 1000× magnification and by immunofluorescence staining to confirm the presence of *C. psittaci* inclusions. For ultrastructural characterization, TEM was performed. Infected cells were fixed in 2.5% glutaraldehyde, post-fixed in 1% osmium tetroxide, dehydrated through a graded ethanol series, embedded in epoxy resin, sectioned, stained with uranyl acetate and lead citrate, and examined using a Hitachi HT7700 transmission electron microscope (Hitachi, Japan) operated at 80 kV.

### Genomic analysis

Raw reads in FASTQ format were subjected to quality control to remove adapter sequences and low-quality bases. Reads were trimmed using an 8-bp sliding window, regions with an average Phred quality score below 20 were removed, terminal bases with quality scores below 20 were trimmed, and paired-end reads shorter than 40 bp after trimming were discarded. The remaining high-quality reads were retained as clean reads for downstream analyses. Each sample generated approximately 8–12 million paired-end reads, corresponding to an average genome coverage depth of 80–120×.

To remove host-derived sequences, clean reads were first aligned to the corresponding host reference genome where applicable, and unmapped reads were retained for pathogen analysis. These unmapped reads were subsequently mapped to the *C. psittaci* strain 6BC reference genome (NC_017287) using BWA-MEM (Houtgast et al., 2018). For genome recovery, pathogen-mapped reads were assembled into contigs using MEGAHIT, and assembled contigs were further compared against the host genome using BLASTn to eliminate residual host contamination.

Consensus genome sequences were generated by mapping clean reads to the best-matching reference genome using BWA-MEM, followed by variant calling with SAMtools (Danecek et al., 2021). Variants were filtered using a minimum sequencing depth of >10× and a minimum base quality of >30, and filtered variants were incorporated into the reference sequence using iVar to generate the final consensus genomes for downstream phylogenetic and comparative genomic analyses. Sequencing and assembly statistics for the 19 genomes, including the numbers of clean reads, average genome coverage, assembled chromosome length, and plasmid size or coverage where applicable, are provided in **Supplementary Table 1**.

After consensus genome sequences were generated, the *ompA* gene sequences of approximately 1050 bp were extracted and used for genotyping and phylogenetic analysis. Multiple sequence alignment was performed using ClustalW in MEGA11 (Tamura et al., 2021). An *ompA*-based phylogenetic tree was reconstructed using the maximum-likelihood method under the Tamura–Nei substitution model with 1000 bootstrap replicates to assess branch support (Nguyen et al., 2015).

Phylogenetic relationships among the study genomes were inferred using a reference-based core-genome SNP approach. Clean reads were mapped to the *C. psittaci* 6BC reference genome using BWA-MEM, and variant calling was performed using the Snippy pipeline, which internally uses SAMtools. A core-genome alignment was generated from all study genomes together with representative publicly available *C. psittaci* genomes retrieved from GenBank. Representative genomes were selected to maximize phylogenetic coverage while minimizing redundancy caused by highly similar strains. Recombinant regions were identified and removed using Gubbins (Croucher et al., 2015) prior to phylogenetic inference. After recombination filtering, a total of 2,487 high-quality core-genome SNPs were identified from a shared core-genome alignment of 964,322 bp. Core SNP sites were extracted using SNP-sites (Page et al., 2016), and a maximum-likelihood phylogenetic tree was reconstructed from the concatenated core-genome SNP alignment with 1000 bootstrap replicates.

Genome annotation was performed using Prokka v1.14 (Seemann, 2014). Acquired antimicrobial resistance genes were screened using ResFinder and the Resistance Gene Identifier (RGI) based on the Comprehensive Antibiotic Resistance Database (CARD) (Alcock et al., 2020). Potential antimicrobial resistance-associated mutations were further assessed by examining the *16S rRNA*, *23S rRNA*, and *rpoB* genes with reference to previously reported resistance-associated loci (Le Roy et al., 2021; Esskhayry et al., 2025). Nucleotide substitutions were identified by multiple sequence alignment in MEGA.

### Intracellular antibiotic inhibition assay

The intracellular inhibitory effects of selected antibiotics against *C. psittaci* isolates were evaluated in McCoy cells. Because whole-genome comparison showed that SZ1 and SZ2 were genetically identical, subsequent antibiotic inhibition assays were performed using SZ1 and SZ3. Cell density was adjusted to 1 × 10⁵ cells/mL, and the multiplicity of infection (MOI) was set to 1. Isolates were adjusted to approximately 1 × 10^6^ inclusion-forming units (IFU)/mL before infection, inoculated into 96-well plates, and incubated for 1 h to allow bacterial entry into host cells.

After infection, the culture medium was replaced with fresh medium containing rifampin, doxycycline, or spectinomycin at final concentrations of 0, 400, 800, 1200, 1600, 2000, 2400, 2800, 3200, and 3600 μg/mL. The same concentration gradient was used for all three antibiotics to allow comparative evaluation under identical experimental conditions. Because standardized susceptibility testing guidelines for *C. psittaci* are currently lacking, a relatively broad concentration range was used to evaluate inhibitory effects on intracellular replication, consistent with previous studies (Huang et al., 2023).

After incubation with antibiotics for 48 h, infected McCoy cells were harvested, and cell pellets were collected for RNA extraction using the QIAGEN RNA Mini Kit (QIAGEN, Hilden, Germany). Extracted RNA was reverse transcribed into cDNA (Shenzhen Aodong Testing Technology Co., Ltd.). Reverse transcription quantitative PCR (RT-qPCR) targeting *ompA* transcripts of *C. psittaci* was performed using a SLAN-96S real-time PCR system (Shanghai, China) to determine cycle threshold (Ct) values at different antibiotic concentrations (Heddema et al., 2006).

Each experimental condition was tested in three independent experiments. Ct values were used as an indirect indicator of intracellular bacterial replication, with lower Ct values indicating higher bacterial loads and higher Ct values indicating stronger inhibition. Dose–response curves were generated to illustrate the relationship between antibiotic concentration and mean Ct value.

To validate the RT-qPCR results using an independent approach, immunofluorescence staining was performed in parallel. Infected cells were fixed, permeabilized, blocked, and incubated with a mouse anti-*C. psittaci* primary antibody, followed by fluorescein isothiocyanate (FITC)-conjugated goat anti-mouse IgG as the secondary antibody. Fluorescence images were captured from three randomly selected microscopic fields per sample using a fluorescence microscope. Images were processed by adjusting brightness and contrast and removing background noise. Fluorescence-positive areas were quantified using ImageJ software (Schneider et al., 2012), and the average fluorescence-positive area was calculated.

Statistical analysis of intracellular antibiotic inhibition was performed using two-way analysis of variance (ANOVA), with isolate and antibiotic concentration as independent factors (Kim, 2014). *Post hoc* multiple comparisons were conducted where appropriate. All statistical analyses were performed using GraphPad Prism.

## Results

### Case presentation

#### Case 1

A 63-year-old woman presented with a one-year history of elevated serum creatinine and a two-day history of fever. At admission, she reported fatigue and lumbar pain, and her body temperature was 39.1 °C. Her medical history included type 2 diabetes mellitus. Administration of diclofenac sodium (75 mg) temporarily reduced her temperature to 36.2 °C. During hospitalization, she developed recurrent fever and respiratory failure, indicating a risk of further clinical deterioration. Acid-fast bacilli smear and blood culture results were negative. BALF tested positive for *C. psittaci* by PCR. Treatment with doxycycline combined with adjunctive antibiotics led to clinical improvement. Although the patient denied direct contact with parrots, epidemiological investigation revealed that a neighbor kept a parrot purchased from a bird market in Futian District; however, direct exposure to the bird could not be confirmed.

#### Case 2

A 69-year-old man presented with cough, rhinorrhea, and a fever of 38.6 °C. Chest radiography showed diffuse patchy opacities in both lungs, whereas the mediastinal structures and cardiac silhouette were unremarkable. The trachea was midline without mediastinal widening or displacement, and the diaphragmatic contours and costophrenic angles were poorly defined. Nasal swabs and BALF were sent to the Shenzhen Center for Disease Control and Prevention for pathogen testing, and both specimens were positive for *C. psittaci*. The patient was treated with doxycycline every 12 h, after which his condition improved. Although he reported no contact with parrots or other pet birds, he had a history of exposure to live ducks before symptom onset.

#### Case 3

A 37-year-old woman presented with chills, headache, fatigue, and a high fever of 39.2 °C. Chest CT revealed persistent inflammatory lesions in the right lower lobe and left upper lobe, together with a small right pleural effusion and a small pericardial effusion. A small nodule was also detected in the left upper lobe, and mild fatty liver was noted. Nasal swabs and BALF were collected and sent to a third-party laboratory (KingMed Diagnostics), where *C. psittaci* was identified. The patient was treated with doxycycline and subsequently improved clinically. She reported no contact with parrots.

#### Case 4

A 40-year-old woman presented to the emergency department with cough, chills, dizziness, and headache. The cough was productive, with a small amount of yellow sputum, and her body temperature was 38.5 °C. Chest imaging revealed patchy consolidation in the middle and lower lobes of the right lung, accompanied by faint ill-defined opacities. A smaller similar lesion was also seen in the left upper lobe, suggesting bilateral pneumonia with more severe involvement of the right lung. In addition, a small high-density focus in the left kidney suggested a renal calculus. During the early stage of hospitalization, the patient received intravenous ambroxol hydrochloride and oral ketotifen, resulting in partial symptomatic relief. Nasal swabs and BALF were collected for pathogen testing and were positive for *C. psittaci*. She was subsequently treated with oral doxycycline hydrochloride, after which her symptoms gradually improved. She reported no contact with parrots.

### Photomicrographs of cells infected with *C. psittaci* isolates

Of the 114 samples tested, 42 were positive for *C. psittaci* DNA by conventional PCR targeting the *ompA* gene. Among the 105 parrot swab samples, the positivity rate was 30.8% (20/65) for cloacal swabs and 40.0% (16/40) for oropharyngeal swabs. Among the five tissue samples collected from the suspected infected parrot, two tested positive for *C. psittaci*, namely tracheal tissue and small-intestinal mucosal tissue. All four BALF samples from laboratory-confirmed psittacosis patients were positive.

**Figure 1:**
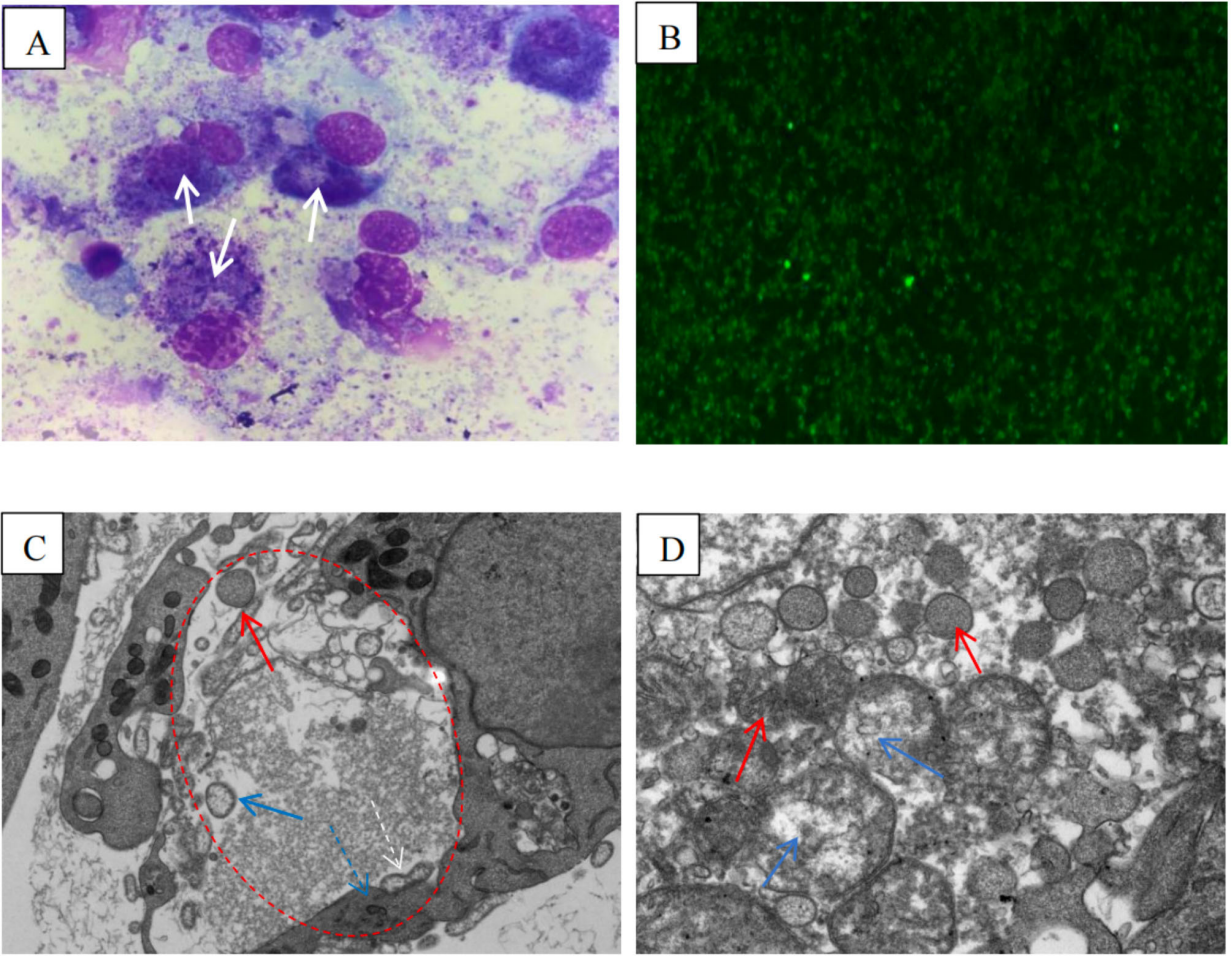
Photomicrographs of cells infected with *C. psittaci.* **(A)** Wright–Giemsa staining of cells infected with *C. psittaci*, observed under light microscopy at 1000× magnification (scale bar = 10 μm). White arrows indicate *C. psittaci* inclusions. **(B)** Immunofluorescence images of infected cells obtained at 4× magnification (scale bar = 500 μm). **(C)** Transmission electron micrograph of an infected cell containing an intracellular inclusion with multiple developmental forms, captured using a Hitachi TEM system (Japan) at 8,000× magnification (80.0 kV; scale bar = 2.0 μm). The red dashed outline marks the inclusion boundary; solid red arrows indicate reticulate bodies (RBs); solid blue arrows indicate elementary bodies (EBs); blue dashed arrows indicate intermediate bodies (IBs); and white dashed arrows indicate dividing RBs. **(D)** Transmission electron micrograph of an infected cell containing an intracellular inclusion with multiple developmental forms, captured at 30,000× magnification (80.0 kV; scale bar = 500 nm). Solid red arrows indicate RBs, and solid blue arrows indicate EBs.

To isolate *C. psittaci*, all 42 PCR-positive samples were inoculated into McCoy cells. After three blind passages, successful isolation was achieved from three samples: BALF from Case 1, tracheal tissue from the suspected infected parrot, and small-intestinal mucosal tissue from the same parrot. Cells inoculated with these three samples produced numerous inclusion bodies on Wright–Giemsa staining (**Figure 1A**) and strong signals on immunofluorescence staining (**Figure 1B**), indicating active intracellular proliferation. TEM further revealed typical elementary bodies (EBs), reticulate bodies (RBs), and intermediate bodies (IBs) with well-defined inclusion membranes (**Figures 1C, D**), supporting successful intracellular propagation. Quantitative PCR of cultured materials showed Ct values of approximately 16, consistent with high bacterial loads. Ultimately, three isolates were obtained: SZ1 from parrot tracheal tissue, SZ2 from parrot small-intestinal mucosal tissue, and SZ3 from BALF of Case 1.

### Maximum-likelihood phylogenetic tree based on the *ompA* gene

**Figure 2:**
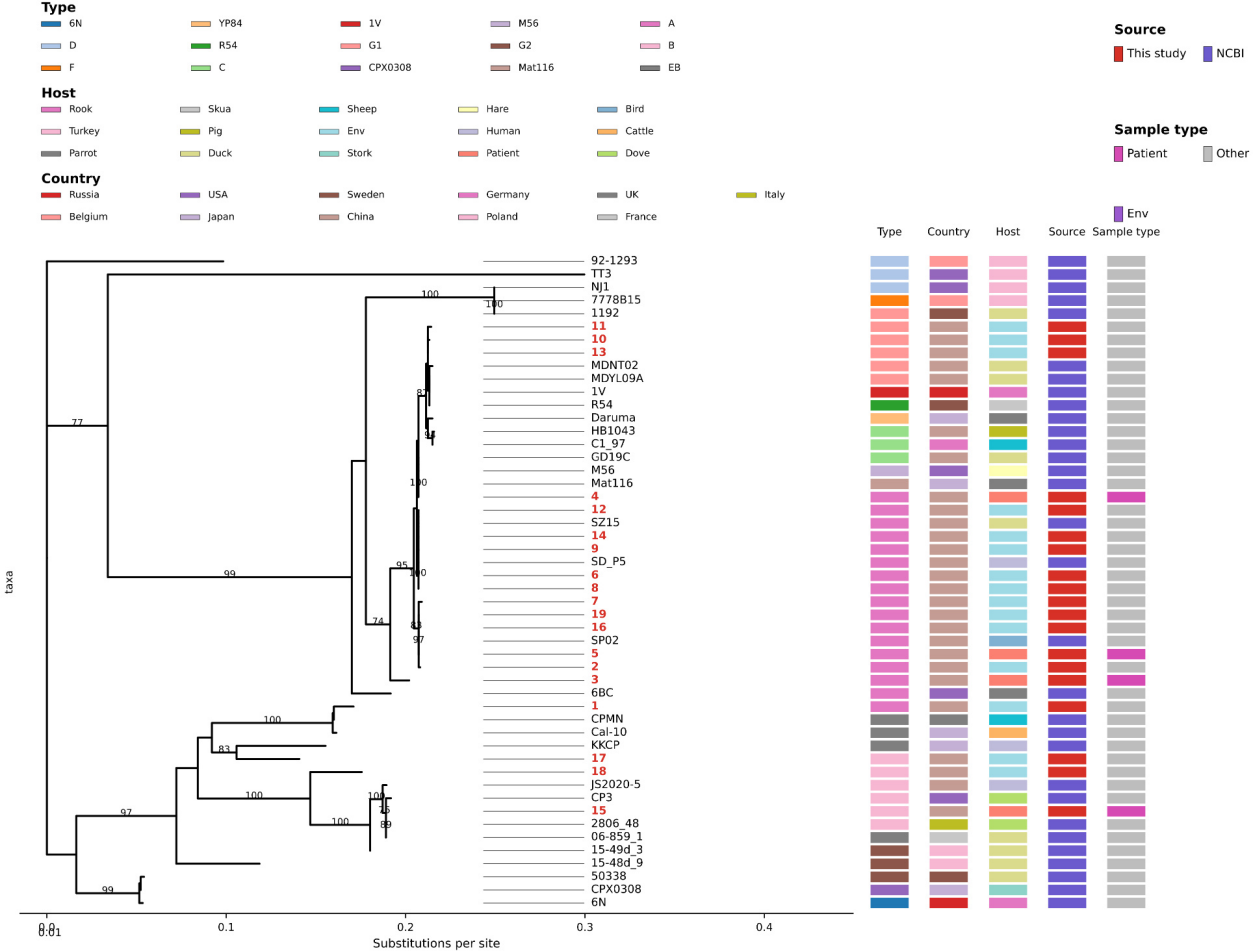
Maximum-likelihood phylogenetic tree of *C. psittaci* based on *ompA* gene sequences. The tree was reconstructed using the maximum-likelihood method. Bootstrap values ≥70 are shown at the nodes. The scale bar indicates the number of nucleotide substitutions per site. Colored annotation bars on the right indicate genotype, country of origin, host, data source, and sample type. Sequences generated in this study are highlighted in red and bold in the tree labels, whereas the Source annotation distinguishes sequences generated in this study from those retrieved from the NCBI database.

**Figure 3:**
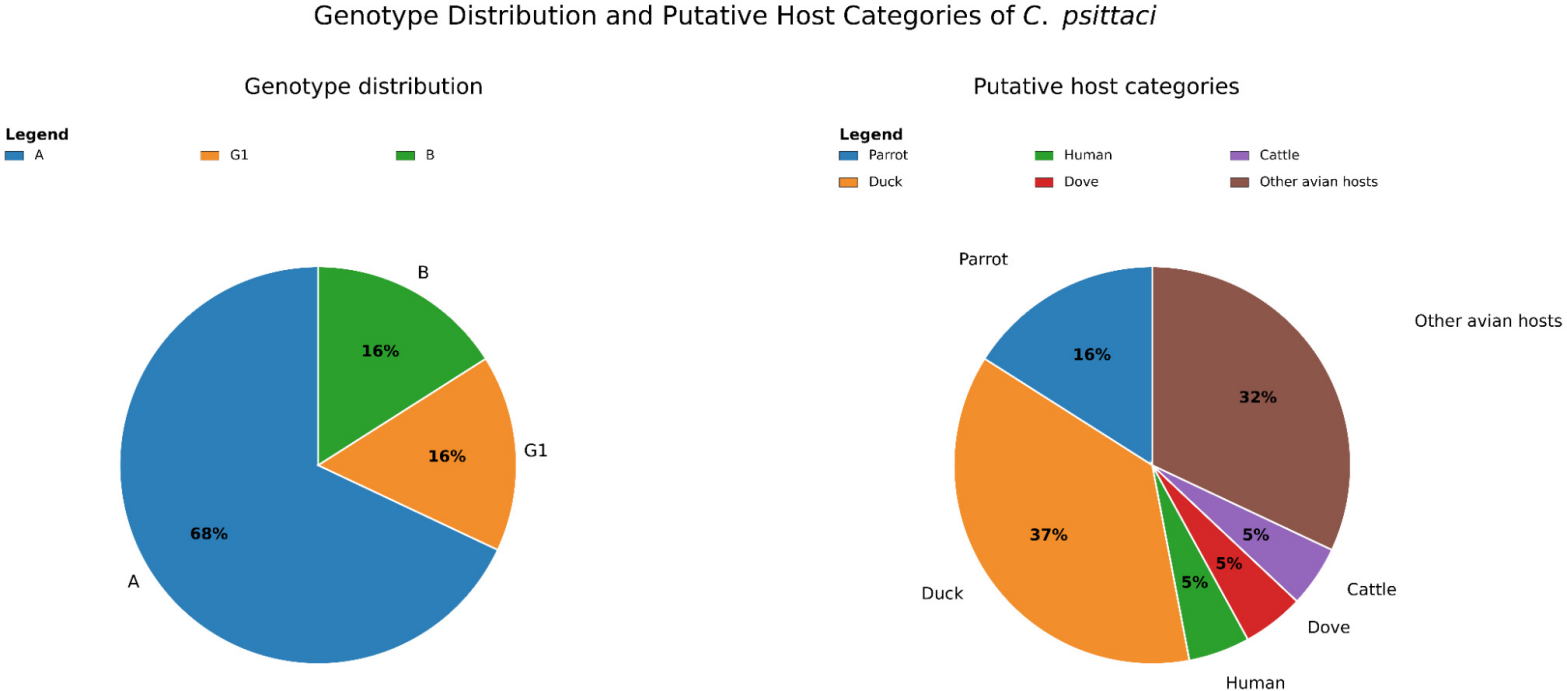
Genotype distribution (left) and putative host categories (right) of the *C. psittaci* sequences analyzed in this study. The genotype pie chart shows the proportions of genotypes A, G1, and B among the analyzed sequences. The host-category pie chart summarizes the inferred host sources based on epidemiological investigation and genomic analysis, including parrot, duck, human, dove, cattle, and other avian hosts.

As shown in **Figure 2**, the *ompA*-based phylogenetic tree indicated that genotype A was predominant, accounting for 68% of the analyzed sequences. As summarized in **Figure 3**, genotypes G1 and B each accounted for 16% of the sequences. Notably, 53% of the analyzed strains originated from avian hosts. Among the four patient-derived samples, three belonged to genotype A based on *ompA* sequence analysis, whereas one belonged to genotype B.

Phylogenetic tree constructed from whole-genome SNP analysis.

**Figure 4:**
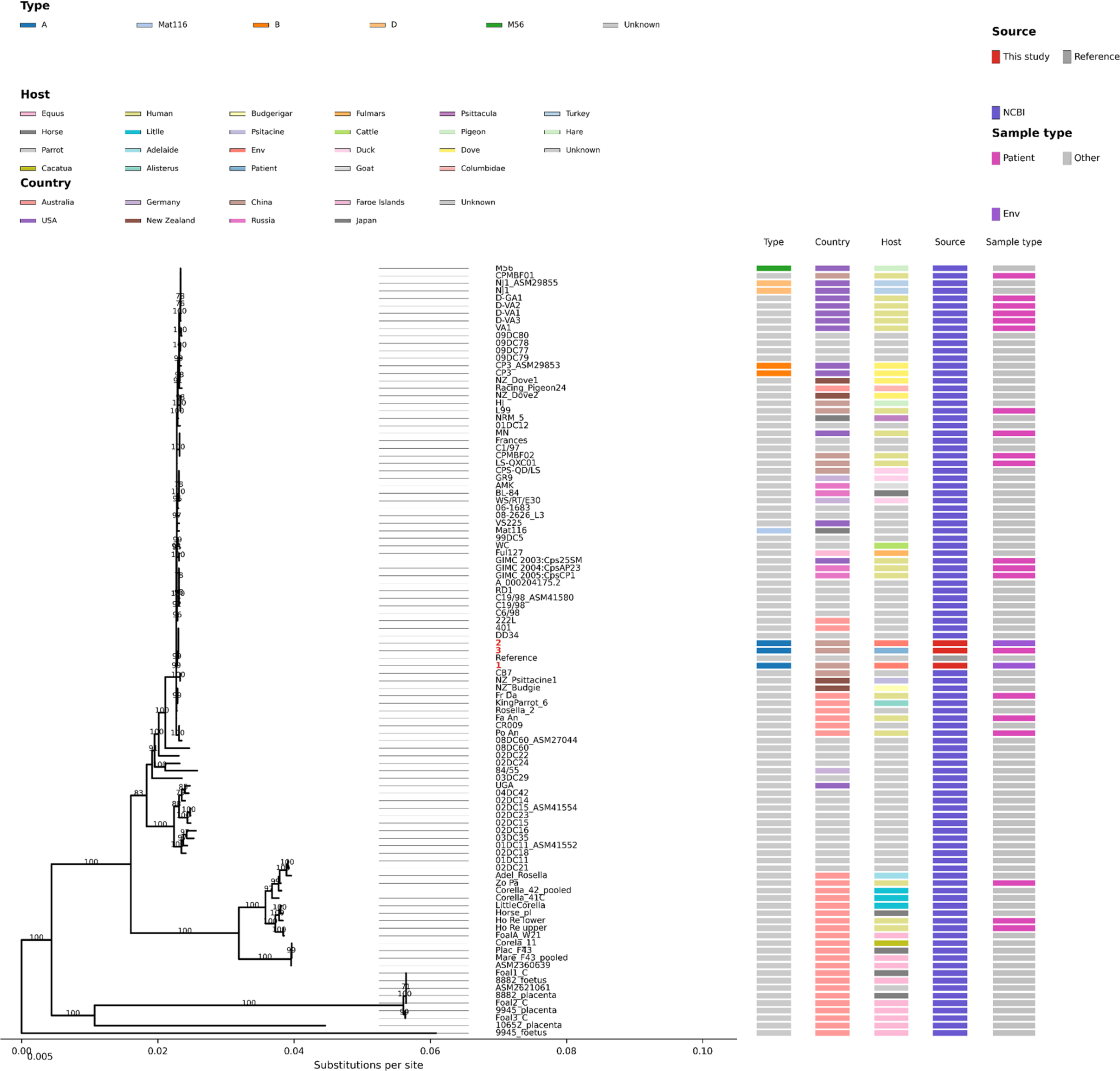
Core-genome SNP-based phylogenetic tree of *C. psittaci* isolates SZ1, SZ2, and SZ3. The tree was reconstructed using the maximum-likelihood method based on high-quality core-genome SNPs identified after recombination filtering with Gubbins. The SNP alignment was generated from a shared core-genome alignment of 964,322 bp. Bootstrap values ≥70 are shown at the nodes, and branch lengths represent genetic distance (substitutions per site). Samples 1, 2, and 3 correspond to SZ1, SZ2, and SZ3, respectively, and are highlighted in red and bold. Colored annotation bars on the right indicate genotype, country of origin, host, data source, and sample type for each isolate. Sequences retrieved from public databases are labeled as NCBI, and the reference genome is indicated separately.

**Figure 4** shows that isolates SZ1, SZ2, and SZ3 clustered most closely with the reference strain 6BC in the core-genome SNP phylogeny. Further analysis showed that strains within this clade were predominantly derived from parrots and other avian hosts. This finding indicates a high degree of genetic similarity between SZ1, SZ2, SZ3, and avian-origin strains in the same clade. Whole-genome comparison additionally showed that isolates SZ1 and SZ2 were genetically identical, indicating that they represent the same *C. psittaci* strain. Accordingly, SZ1 was selected to represent the parrot-derived isolate in subsequent antibiotic inhibition assays, and SZ3 was used as the human-derived comparator.

### Analysis of antimicrobial resistance-associated genes and mutations

Screening using ResFinder and the Resistance Gene Identifier (RGI) based on the Comprehensive Antibiotic Resistance Database (CARD) showed that none of the 19 C*. psittaci* genomes carried known acquired antimicrobial resistance genes. Specifically, ResFinder detected no acquired resistance determinants, suggesting the absence of typical exogenous resistance elements such as β-lactamase genes or efflux-associated determinants. Likewise, RGI analysis based on CARD did not identify any definitive resistance genes or established resistance-associated mutations (Bortolaia et al., 2020). Multiple sequence alignment of *16S rRNA* gene sequences in MEGA (Kumar et al., 2018) revealed nucleotide substitutions at positions A1191G in 16 of 19 samples (84.2%) and C1192G in 17 of 19 samples (89.5%), whereas no mutation was detected at position C1193G. In addition, alignment of the *rpoB* gene showed that none of the isolates carried the AUG→AUC substitution corresponding to the Met515→Ile mutation. The point mutations identified in the 16S rRNA and rpoB genes are summarized in **Table 1**.

**Table 1:** Point mutations identified in the *16S rRNA* and *rpoB* genes.

Gene	Locus	Nucleotide mutation	Amino acid change	Number of mutant samples	Proportion of mutant samples
*16S rRNA*	1191	A1191G	–	1, 2, 3, 4, 5, 6, 7, 9, 11, 12, 13, 14, 15, 16, 18, 19	84.2% (16/19)
*16S rRNA*	1192	C1192G	–	1, 2, 3, 4, 5, 6, 7, 9, 10, 11, 12, 13, 14, 15, 16, 18, 19	89.5% (17/19)
*16S rRNA*	1193	C1193G	–	None	0% (0/19)
*rpoB*	515	G515C	Met515 → Ile	None	0% (0/19)

### Intracellular antibiotic inhibition analysis

#### Intracellular response to rifampin

**Figure 5:**
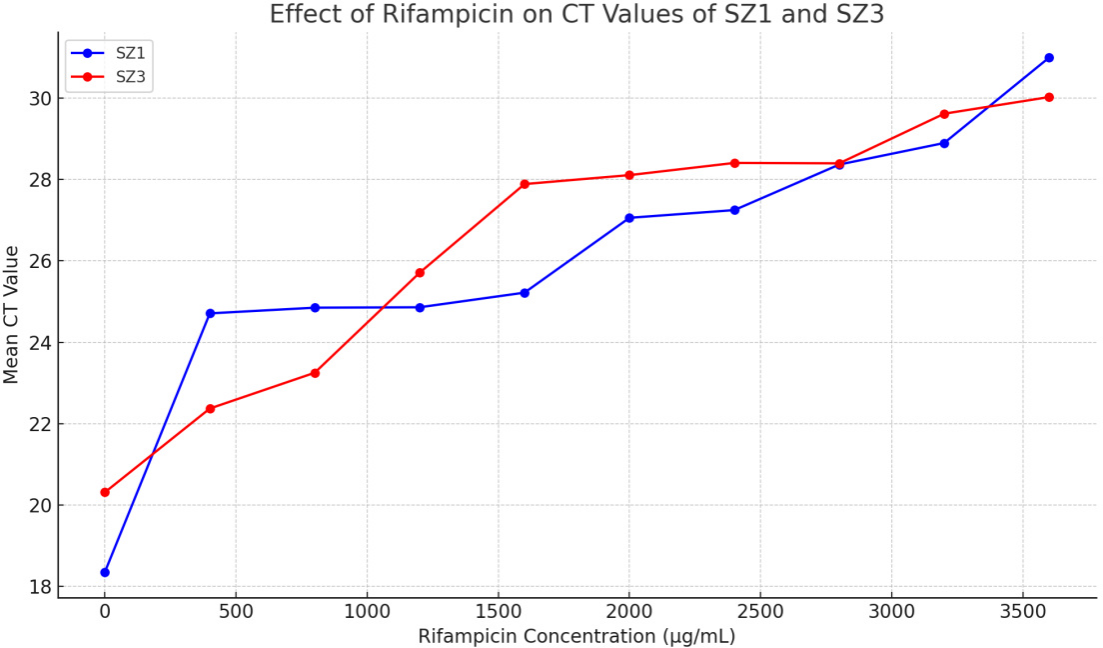
Effect of rifampin concentration on Ct values in *C. psittaci* isolates SZ1 and SZ3. The x-axis shows rifampin concentration (μg/mL), and the y-axis shows Ct values obtained by RT-qPCR. Data are presented as mean ± standard deviation from three independent experiments.

With increasing rifampin concentrations, the Ct values of both SZ1 and SZ3 gradually increased, indicating reduced intracellular replication at higher drug exposure. For SZ1, a marked increase in Ct values was observed at concentrations above 1600 μg/mL, whereas SZ3 showed a similar inhibitory response at concentrations above 1200 μg/mL. At rifampin concentrations exceeding 3200 μg/mL, Ct values reached or exceeded 30, indicating negative detection and suggesting complete inhibition of *C. psittaci* replication (**Figure 5**).

**Figure 6:**
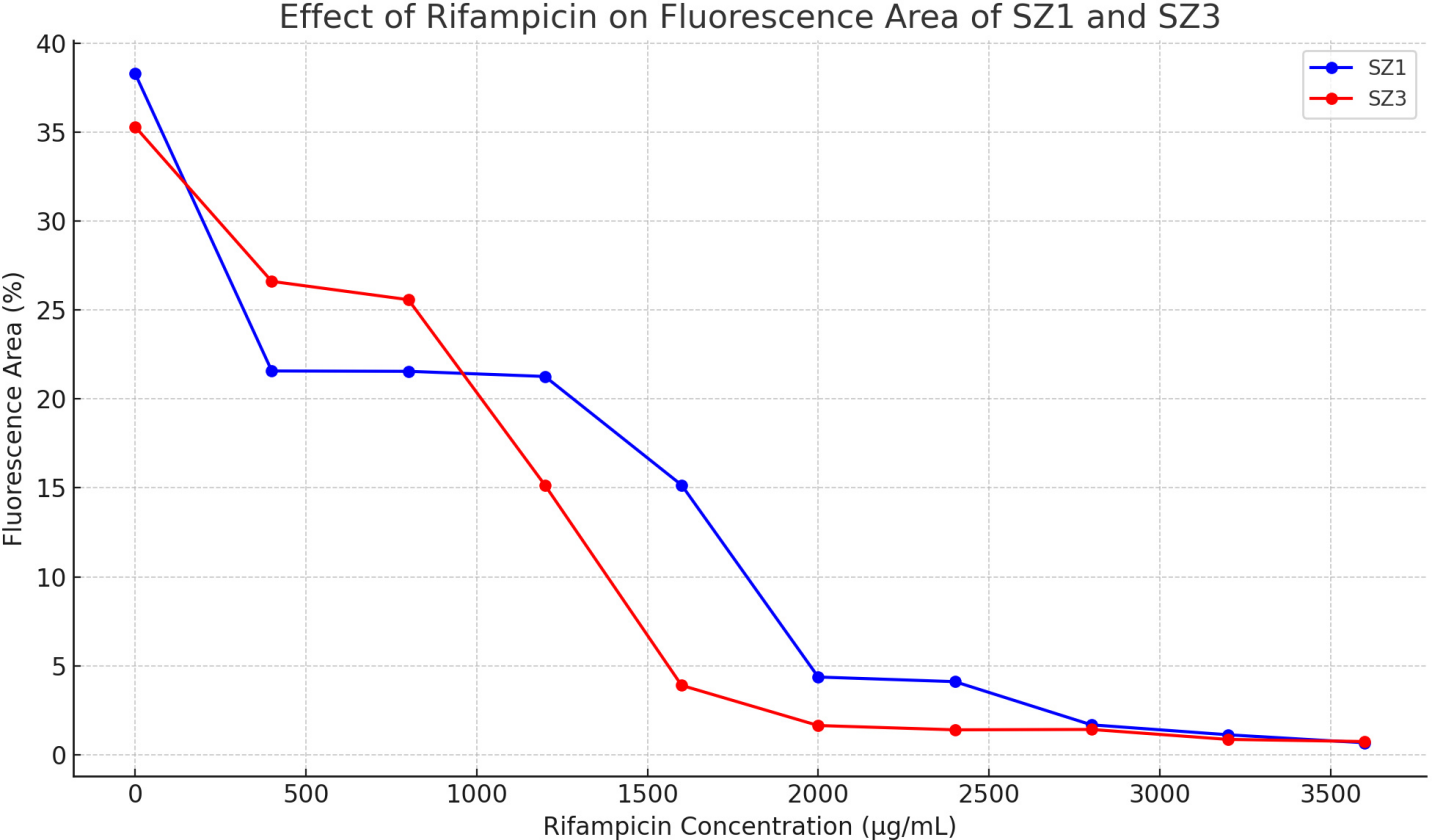
Effect of rifampin concentration on fluorescence-positive area in *C. psittaci* isolates SZ1 and SZ3. The x-axis shows rifampin concentration (μg/mL), and the y-axis shows fluorescence-positive area (%), calculated as the proportion of positive fluorescence signal relative to the total field of view. Data are presented as mean ± standard deviation from three independent immunofluorescence assays.

Consistently, immunofluorescence analysis showed a progressive decrease in fluorescence intensity and fluorescence-positive area with increasing rifampin concentrations. At 3200 μg/mL, the fluorescence-positive area decreased to less than 1%, further supporting near-complete inhibition of bacterial growth (**Figure 6**).

Two-way ANOVA showed that rifampin concentration significantly affected Ct values (F = 284.46, p < 0.0001). A significant difference between isolates SZ1 and SZ3 was also observed (F = 7.66, p = 0.0085). In addition, a significant isolate × concentration interaction was detected (F = 15.96, p < 0.0001), indicating that the two isolates displayed different response patterns across the rifampin concentration gradient (**Table 2**). The immunofluorescence results were consistent with the RT-qPCR data, supporting the robustness of the comparative phenotypic assessment.

**Table 2:** Two-way ANOVA analysis of rifampin susceptibility in *C. psittaci* isolates SZ1 and SZ3.

Source	df	F	p-value
Isolate	1	7.66	0.0085
Concentration	9	284.46	<0.0001
Isolate × Concentration	9	15.96	<0.0001

#### Intracellular response to doxycycline

**Figure 7:**
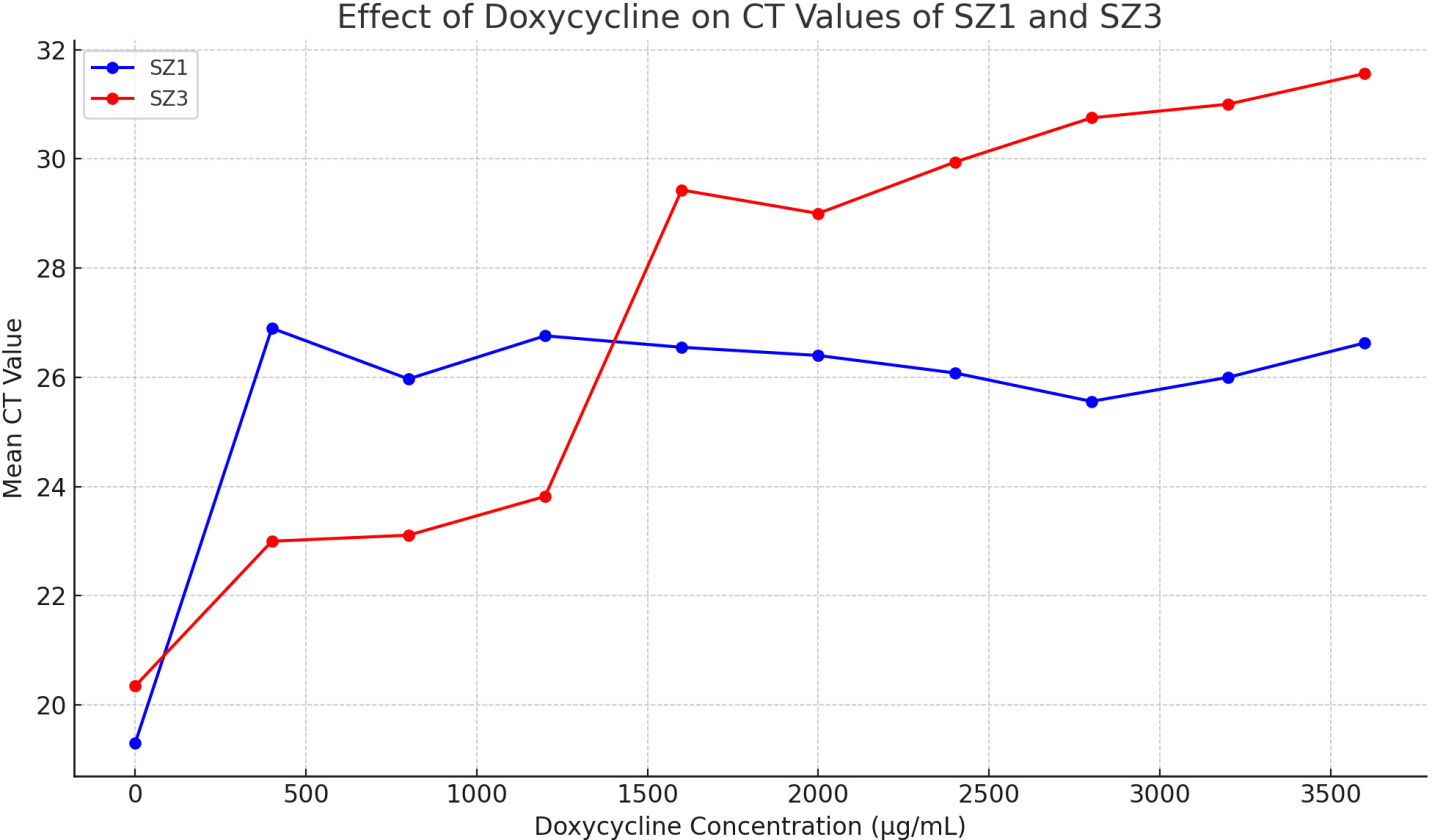
Effect of doxycycline concentration on Ct values in *C. psittaci* isolates SZ1 and SZ3. The x-axis shows doxycycline concentration (μg/mL), and the y-axis shows Ct values obtained by RT-qPCR. Data are presented as mean ± standard deviation from three independent experiments.

For isolate SZ1, the baseline Ct value in the absence of doxycycline was 19.31. As doxycycline concentration increased, Ct values remained relatively stable at approximately 26, with no marked additional increase even at higher concentrations. In contrast, isolate SZ3 showed a baseline Ct value of 20.35 without doxycycline treatment. When the doxycycline concentration exceeded 1200 μg/mL, a clear inhibitory effect was observed in SZ3, with progressively increasing Ct values. At concentrations ≥2000 μg/mL, Ct values reached or exceeded 30, indicating negative detection and suggesting complete inhibition of *C. psittaci* replication (**Figure 7**).

**Figure 8:**
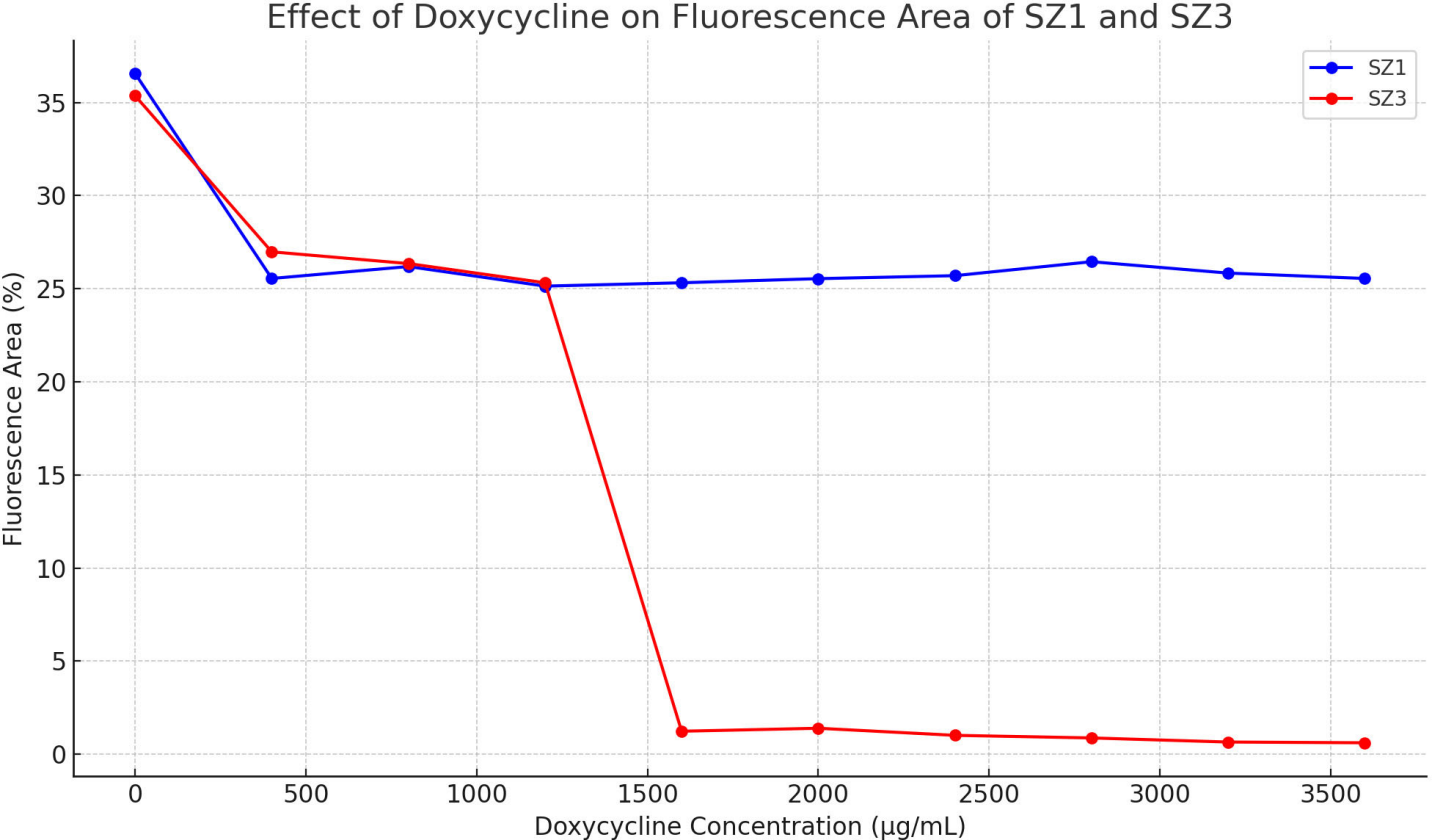
Effect of doxycycline concentration on fluorescence-positive area in *C. psittaci* isolates SZ1 and SZ3. The x-axis shows doxycycline concentration (μg/mL), and the y-axis shows fluorescence-positive area (%), calculated as the proportion of positive fluorescence signal relative to the total field of view. Data are presented as mean ± standard deviation from three independent immunofluorescence assays.

Measurements of fluorescence intensity and fluorescence-positive area further supported these findings. For SZ1, increasing doxycycline concentrations did not result in substantial changes, and the fluorescence-positive area remained around 26%. By contrast, SZ3 showed a progressive decrease in both fluorescence intensity and fluorescence-positive area with increasing doxycycline concentration. At 2000 μg/mL, the fluorescence-positive area fell below 2%, consistent with near-complete growth inhibition. The doxycycline concentration-dependent changes in the fluorescence-positive area are shown in **Figure 8**.

Two-way ANOVA showed that doxycycline concentration significantly affected Ct values (F = 77.19, p = 1.93 × 10^-22^). A significant difference between isolates SZ1 and SZ3 was also observed (F = 60.47, p = 1.59 × 10^-9^). Moreover, a significant isolate × concentration interaction was detected (F = 30.94, p = 2.73 × 10^-15^), indicating distinct response patterns between the two isolates across the doxycycline gradient (**Table 3**). The immunofluorescence findings were consistent with the RT-qPCR data, supporting the reliability of the comparative assessment.

**Table 3:** Two-way ANOVA analysis of doxycycline susceptibility in *C. psittaci* isolates SZ1 and SZ3.

Source	df	F	p-value
Isolate	1	60.47	1.59 × 10^-9^
Concentration	9	77.19	1.93 × 10^-22^
Isolate × Concentration	9	30.94	2.73 × 10^-15^

#### Intracellular response to spectinomycin

With increasing spectinomycin concentrations, Ct values of both isolates gradually increased, indicating reduced intracellular replication under higher drug exposure. For isolate SZ1, the baseline Ct value in the absence of spectinomycin was 18.35. Ct values remained relatively stable at approximately 20 when concentrations were ≤800 μg/mL, whereas concentrations above 3200 μg/mL resulted in Ct values ≥30, indicating negative detection and suggesting complete inhibition of bacterial replication.

**Figure 9:**
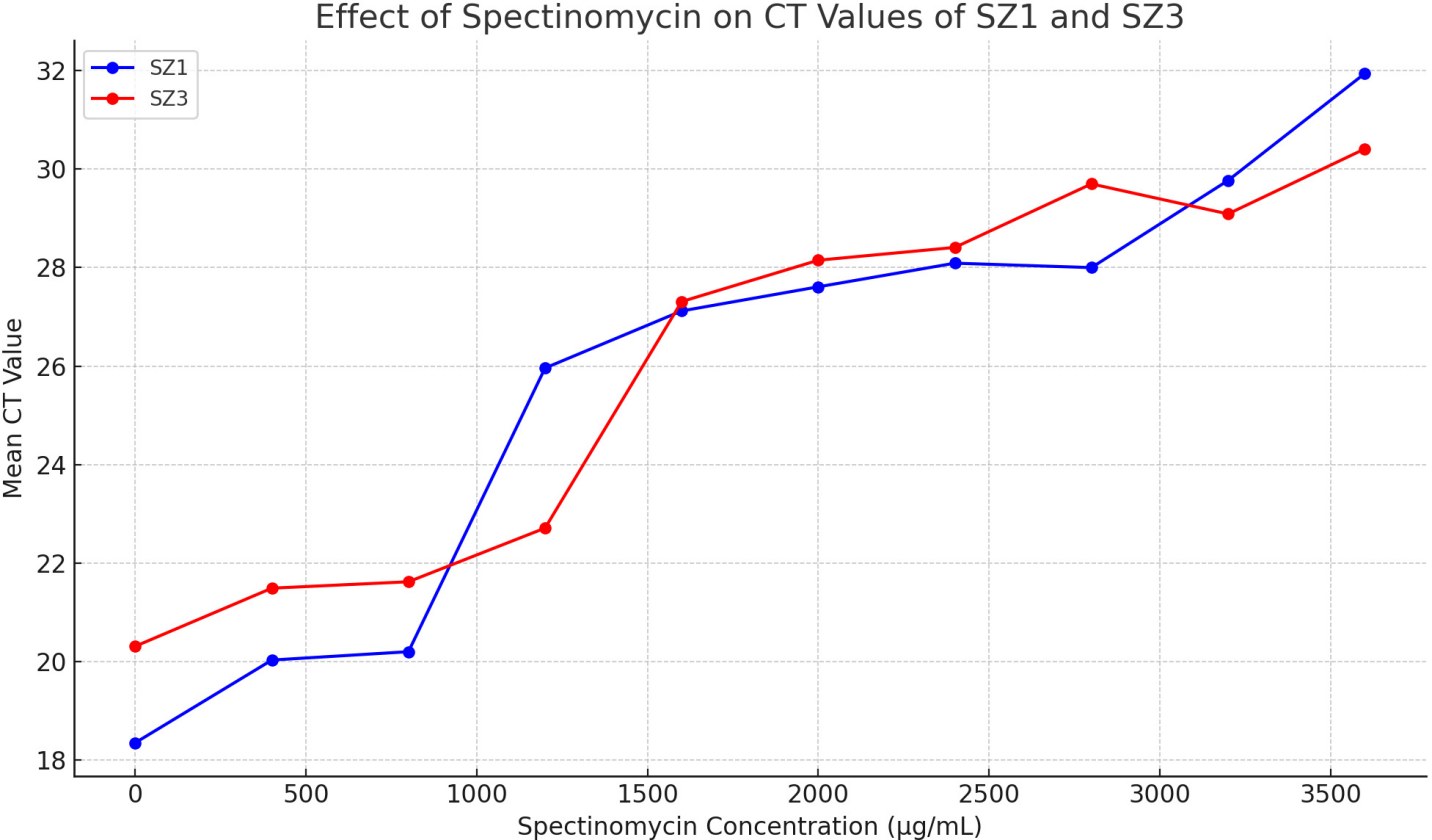
Effect of spectinomycin concentration on Ct values in *C. psittaci* isolates SZ1 and SZ3. The x-axis shows spectinomycin concentration (μg/mL), and the y-axis shows Ct values obtained by RT-qPCR. Data are presented as mean ± standard deviation from three independent experiments.

For isolate SZ3, the baseline Ct value without spectinomycin was 20.31. Ct values remained stable at approximately 21 when concentrations were ≤1200 μg/mL, but increased markedly at higher concentrations, reaching ≥30 when concentrations exceeded 2800 μg/mL, again suggesting complete inhibition of *C. psittaci* replication (**Figure 9**).

**Figure 10:**
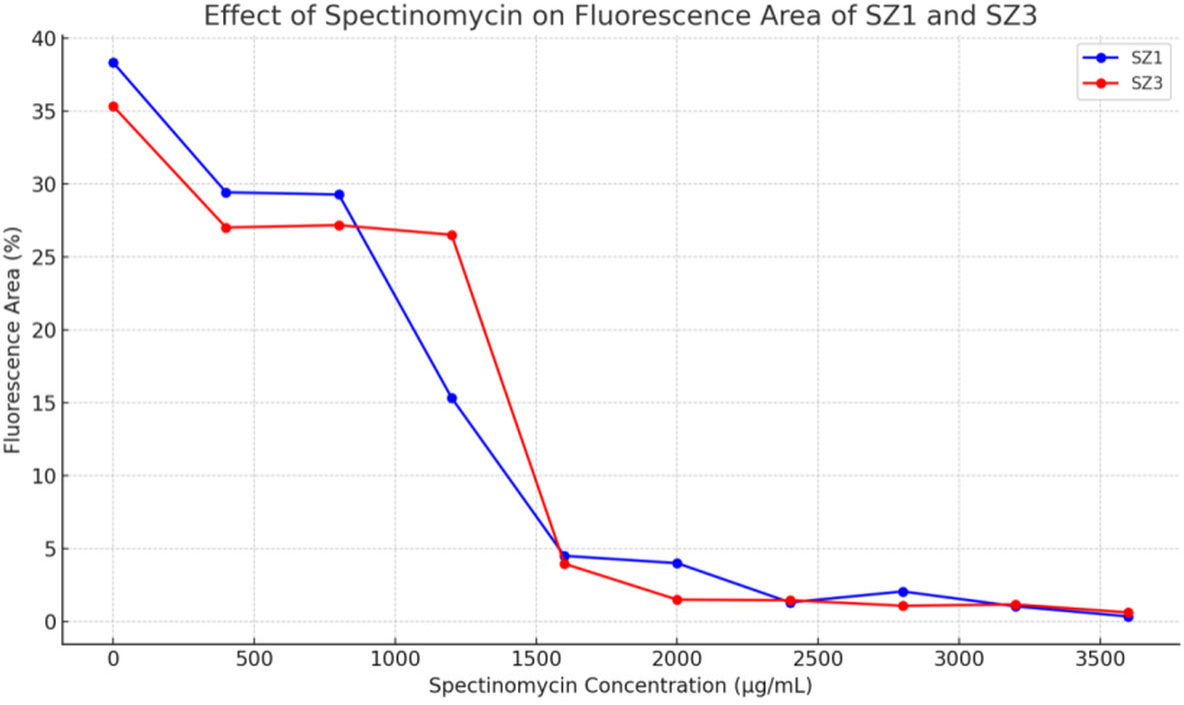
Effect of spectinomycin concentration on fluorescence-positive area in *C. psittaci* isolates SZ1 and SZ3. The x-axis shows spectinomycin concentration (μg/mL), and the y-axis shows fluorescence-positive area (%), calculated as the proportion of positive fluorescence signal relative to the total field of view. Data are presented as mean ± standard deviation from three independent immunofluorescence assays.

Immunofluorescence analysis further supported these observations. In SZ1, fluorescence intensity and fluorescence-positive area remained around 27% when spectinomycin concentrations were ≤800 μg/mL, but gradually declined at higher concentrations, decreasing to below 1% at concentrations >3200 μg/mL. Similarly, in SZ3, the fluorescence-positive area remained around 27% at concentrations ≤1200 μg/mL but decreased substantially at higher concentrations, eventually falling below 1% at concentrations >3200 μg/mL (**Figure 10**).

Two-way ANOVA showed that spectinomycin concentration significantly affected Ct values (F = 185.30, p < 0.0001). However, no overall difference between isolates SZ1 and SZ3 was observed (F = 0.377, p = 0.543). A significant isolate × concentration interaction was detected (F = 6.643, p < 0.0001), indicating different response patterns across the concentration gradient (**Table 4**). The immunofluorescence results were concordant with the RT-qPCR findings, supporting the reliability of the combined phenotypic assessment.

**Table 4:** Two-way ANOVA analysis of spectinomycin susceptibility in *C. psittaci* isolates SZ1 and SZ3.

Source	df	F	p-value
Isolate	1	0.377	0.543
Concentration	9	185.300	<0.0001
Isolate × Concentration	9	6.643	<0.0001

## Discussion

In China, the rapid expansion of the poultry industry and the increasing trade in captive birds have created favorable conditions for the maintenance and spread of *C. psittaci*, making this pathogen an emerging public health concern (Huang et al., 2023). Live-bird markets and breeding facilities, which are characterized by high bird density, frequent animal movement, and close human–bird interactions, provide favorable settings for pathogen circulation and potential cross-species transmission (Jones et al., 2013). In addition to direct contact with infected birds, *C. psittaci* may also be transmitted through inhalation of aerosolized respiratory secretions, dried feces, contaminated feathers, or dust, thereby increasing the risk of infection in bird-associated environments where humans and animals interact closely (Dickx et al., 2010).

In the present study, 42 of 114 samples were positive for *C. psittaci* DNA by conventional PCR, indicating the presence of this pathogen in both human clinical and parrot-associated samples in Shenzhen. Among the 105 parrot swab samples, oropharyngeal swabs showed a numerically higher positivity rate than cloacal swabs. This observation may suggest that oropharyngeal swabs could be useful for detecting *C. psittaci* in parrots. However, because no statistical comparison was performed and the sample size was limited, this finding should be interpreted cautiously and requires further validation in larger studies (Ayllón et al., 2025). By contrast, the relatively lower positivity rate in cloacal swabs may be related to intermittent fecal shedding, lower bacterial burden in the gastrointestinal tract, or differences in sampling timing, although these possibilities require further investigation.

Among the five tissue samples collected from one suspected infected parrot, tracheal tissue and small-intestinal mucosal tissue tested positive for *C. psittaci*. Detection of the pathogen in these two tissues suggests that both respiratory and intestinal tissues may be involved in infection in this individual bird (Zhang et al., 2024). However, because the tissue findings were obtained from only one suspected infected parrot, they should not be overinterpreted as evidence of a general tissue tropism pattern. Together with the positive oropharyngeal and cloacal swab results from market parrots, these observations suggest that infected parrots may contribute to environmental contamination and potential zoonotic exposure in settings with frequent human–bird contact (Miyashita et al., 1993).

Among the four laboratory-confirmed human cases included in this study, three were associated with *C. psittaci* genotype A based on *ompA* sequence analysis, whereas one was associated with genotype B. Epidemiological investigation suggested possible exposure routes in some cases. Case 1, which was associated with genotype A, had a possible indirect exposure to a parrot in the residential environment, whereas Case 2, which was associated with genotype B, had a history of exposure to live ducks before symptom onset. Although direct transmission links could not be confirmed, these observations are consistent with the recognized zoonotic transmission patterns of *C. psittaci*, in which infection is often associated with exposure to infected birds or contaminated environments (Spoorenberg et al., 2016).

*ompA*-based genotyping further showed that genotype A was predominant among the strains analyzed in this study. Notably, the human-derived isolate SZ3 and the parrot-derived isolate SZ1 both belonged to genotype A and clustered closely with avian-origin strains in the phylogenetic analyses. This close genetic relationship suggests a possible epidemiological connection between human and avian sources and highlights the potential role of parrots as reservoirs of zoonotic infection (Vanrompay et al., 2007). Previous genomic studies have likewise shown that *ompA* genotype A is frequently associated with human psittacosis and cross-species transmission (Hogerwerf et al., 2020). Nevertheless, because only a limited number of isolates were successfully recovered and direct exposure evidence was incomplete, a bird-to-human transmission event should be considered plausible rather than definitively proven.

The simultaneous detection of *C. psittaci* in human clinical samples and parrot-associated samples underscores the importance of integrated surveillance at the human–animal interface. Settings characterized by frequent human–bird contact, including live-bird markets, pet shops, and breeding facilities, may represent key environments for zoonotic spillover (Fauziah et al., 2024). Strengthening surveillance systems and implementing improved biosecurity measures in these settings may therefore help reduce the risk of psittacosis outbreaks and limit the spread of *C. psittaci* in urban ecosystems (Sun et al., 2025).

In the present study, intracellular antibiotic inhibition assays were used as a comparative phenotypic approach to evaluate the responses of selected *C. psittaci* isolates to rifampin, doxycycline, and spectinomycin. Because standardized susceptibility testing guidelines for *C. psittaci* are currently lacking, the present findings should be interpreted as comparative intracellular inhibition results rather than formal minimum inhibitory concentration determinations.

For rifampin, both SZ1 and SZ3 showed concentration-dependent increases in Ct values and decreases in fluorescence-positive areas, indicating reduced intracellular replication at higher drug concentrations. Two-way ANOVA demonstrated that rifampin concentration significantly affected Ct values, and a significant isolate × concentration interaction was also observed, indicating concentration-dependent differences in response between SZ1 and SZ3. However, genomic analysis did not identify known *rpoB* resistance-associated mutations, and both qPCR and immunofluorescence data indicated effective inhibition at higher concentrations. These results are consistent with previous reports suggesting that *C. psittaci* generally remains susceptible to rifampin (Benamri et al., 2021). Taken together, the absence of known resistance-associated mutations and the observed phenotypic inhibition suggest that rifampin retained intracellular inhibitory activity against these isolates under the experimental conditions used here.

The widespread use and misuse of antibiotics may contribute to the emergence of *C. psittaci* strains with altered antimicrobial susceptibility, which represents an increasing public health concern (Zhou et al., 2024). For doxycycline, SZ1 and SZ3 displayed distinct response patterns across the tested concentration gradient. Consistent with the Ct curves and immunofluorescence data, doxycycline showed a less pronounced inhibitory effect against SZ1 than against SZ3 under the present experimental conditions. This observation suggests reduced doxycycline susceptibility rather than confirmed resistance. Because doxycycline remains the first-line treatment for psittacosis, the emergence of strains with reduced susceptibility could complicate clinical management and increase the public health risk associated with zoonotic transmission (Butaye et al., 1997). These findings emphasize the need for continued surveillance and for further investigation into the mechanisms underlying altered doxycycline susceptibility in *C. psittaci (*Verminnen et al., 2008). Prudent antimicrobial use and routine monitoring of susceptibility patterns are therefore warranted.

For spectinomycin, two-way ANOVA demonstrated significant effects of antibiotic concentration and a significant isolate × concentration interaction, whereas no overall difference was observed between SZ1 and SZ3. Phenotypically, both isolates displayed concentration-dependent inhibition patterns. However, genomic analysis identified conserved substitutions at previously reported resistance-associated sites in the *16S rRNA* gene despite the absence of known acquired resistance genes. This apparent genotype–phenotype discordance suggests that these substitutions alone may be insufficient to confer measurable resistance under the present experimental conditions, or that additional genetic and physiological factors may modulate the final phenotype (Le Roy et al., 2021). It is also possible that very high spectinomycin concentrations affected host-cell viability and thereby indirectly influenced intracellular bacterial replication (Butler et al., 2018). Because host-cell cytotoxicity was not directly assessed in this study, this possibility remains speculative and should be addressed in future work. Overall, these findings highlight the complexity of interpreting antimicrobial susceptibility in obligate intracellular pathogens and underscore the importance of integrating phenotypic testing with genomic evidence when evaluating potential resistance in *C. psittaci (*Huang et al., 2023).

To prevent and control psittacosis outbreaks, integrated surveillance of *C. psittaci* should be strengthened. Targeted education and training for bird breeders, live-bird market personnel, and other stakeholders in infection prevention and control and antimicrobial stewardship are also essential (Beeckman and Vanrompay, 2009). Reducing antibiotic misuse may help limit the emergence and spread of strains with reduced susceptibility, thereby lowering zoonotic transmission risk and improving treatment outcomes.

This study has several limitations. First, although all PCR-positive samples were inoculated into McCoy cells, only three isolates were successfully recovered, limiting the representativeness of the phenotypic comparison. Second, tissue samples were obtained from only one suspected infected parrot; therefore, the tissue distribution findings should not be generalized to broader parrot populations. Third, antibiotic effects were evaluated using a combined RT-qPCR and immunofluorescence-based intracellular inhibition approach rather than standardized inclusion-counting assays; the results should therefore be interpreted as comparative intracellular inhibition data rather than formal minimum inhibitory concentration determinations. Fourth, host-cell cytotoxicity at high antibiotic concentrations was not assessed. Future studies incorporating standardized chlamydial susceptibility assays, larger strain collections, host-cell viability assays, and complementary functional experiments will be necessary to further validate these findings.

## Data Availability

The datasets presented in this study can be found in online repositories. The names of the repository/repositories and accession number(s) can be found in the article/**Supplementary Material**. Additional data supporting the conclusions of this article are included within the article and its **Supplementary Material**.

## Supplementary material

Supplementary Table 1 Sequencing and assembly statistics for the 19 C. psittaci genomes analyzed in this study.
